# Transvaginal Evisceration of the Small Bowel More Than 15 Years After Abdominal Hysterectomy and Vaginal Surgery

**DOI:** 10.7759/cureus.13955

**Published:** 2021-03-17

**Authors:** Amy Kar Yan Chan, Oluwaseun Oluwajobi, Aisha Ehsan, Farshad Tahmasebi

**Affiliations:** 1 Obstetrics and Gynaecology, Russells Hall Hospital, The Dudley Group NHS Foundation Trust, Dudley, GBR; 2 General and Colorectal Surgery, Russells Hall Hospital, The Dudley Group NHS Foundation Trust, Dudley, GBR; 3 General Surgery, Russells Hall Hospital, The Dudley Group NHS Foundation Trust, Dudley, GBR

**Keywords:** transvaginal evisceration, abdominal surgery, gynaecology emergency

## Abstract

Transvaginal evisceration of the intra-abdominal organs is a rare emergency event. In this paper, we discuss the case of a 97-year-old female who presented to the emergency department due to abdominal pain and a large prolapse with visible extrusion of the small bowel per vagina. Past surgical history was significant for a total abdominal hysterectomy and surgical repair for pelvic organ prolapse; both performed more than 15 years prior to the patient's current presentation. The eviscerated bowel was initially reduced through a vaginal vault defect into the abdominal cavity. A lower midline laparotomy was undertaken for further assessment, and the vault defect was closed by transabdominal repair with no evident compromise to bowel function. We suggest that a multidisciplinary approach to prompt examination and management by gynaecology and general surgery is vital in reducing the risk of morbidity and mortality.

## Introduction

Transvaginal evisceration describes the extrusion of intra-abdominal viscera through the vaginal vault and is an emergency. It is a rare event, and, of those reported, the small bowel is the most commonly involved organ. Relatively few cases have been published in the medical literature, and the exact incidence is difficult to determine. Aetiology is also unclear, but there are common risk factors that have been noted. Most cases occur in post-menopausal women of advanced age who have undergone previous hysterectomy and vaginal surgery [1]. The mean age of affected patients is found to be 62 years [2]. Precipitating factors for vaginal vault dehiscence and subsequent organ evisceration include suddenly raised intra-abdominal pressure and vaginal trauma in the immediate period after vaginal surgery, for example, from instrumentation or sexual intercourse [1]. Prompt management is required to prevent irreversible compromise of organ function and significant morbidity [1].

We present a case of a transvaginal evisceration of the small bowel in a patient who underwent abdominal hysterectomy 17 years ago and had a previous history of vaginal repair for pelvic organ prolapse.

## Case presentation

**Figure 1:**
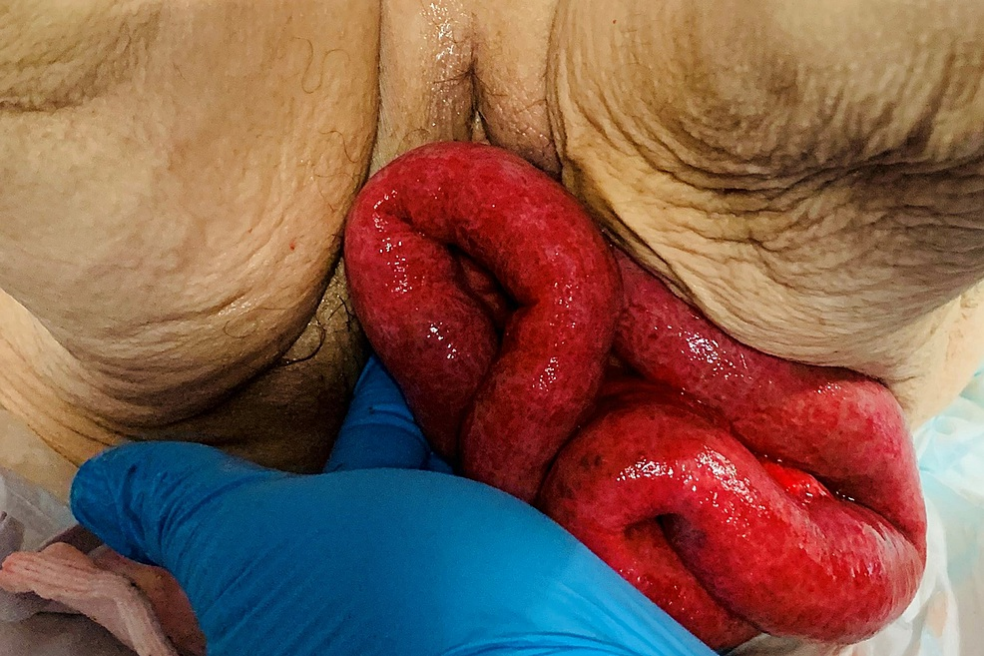
Transvaginal evisceration of the small bowel loops at presentation, supported by the gloved hand of the attending clinician.

A 97-year-old woman presented to the emergency department at a district general hospital with severe abdominal pain and vomiting. The patient was referred to the gynaecology team due to examination revealing a large vault prolapse, with visible loops of bowel extruding from the vaginal introitus (Figure 1). Multiple loops of the small bowel and abdominal mesentery were involved. Urgent assessment and management were performed together by gynaecology and general surgery. The exposed loops of the bowel were pink in colour and appeared viable; these were immediately covered with warm, moistened gauze. A digital rectal exam confirmed that there was no involvement of the rectum.

The patient had been recently discharged from a hospital following a hemiarthroplasty for a fractured neck of the femur and was being cared for at a rehabilitation facility. On the morning of admission, the patient reported feeling unwell, experiencing abdominal discomfort, and falling. Her medical history included chronic issues with constipation, diverticular disease, chronic kidney disease, hypertension, visual impairment, and age-related cerebrovascular changes.

The patient had a parity of one and had undergone total abdominal hysterectomy and bilateral salpingo-oophorectomy 17 years earlier for a benign indication. Furthermore, surgical repair of a posterior vaginal wall prolapse had been carried out 15 years earlier. The patient’s next of kin relayed that despite having recurrence of prolapse, she was deemed an unsuitable candidate for further surgical repair. Unfortunately, historical details of previous gynaecological surgeries and any follow-up consultations are not available to the authors.

**Figure 2:**
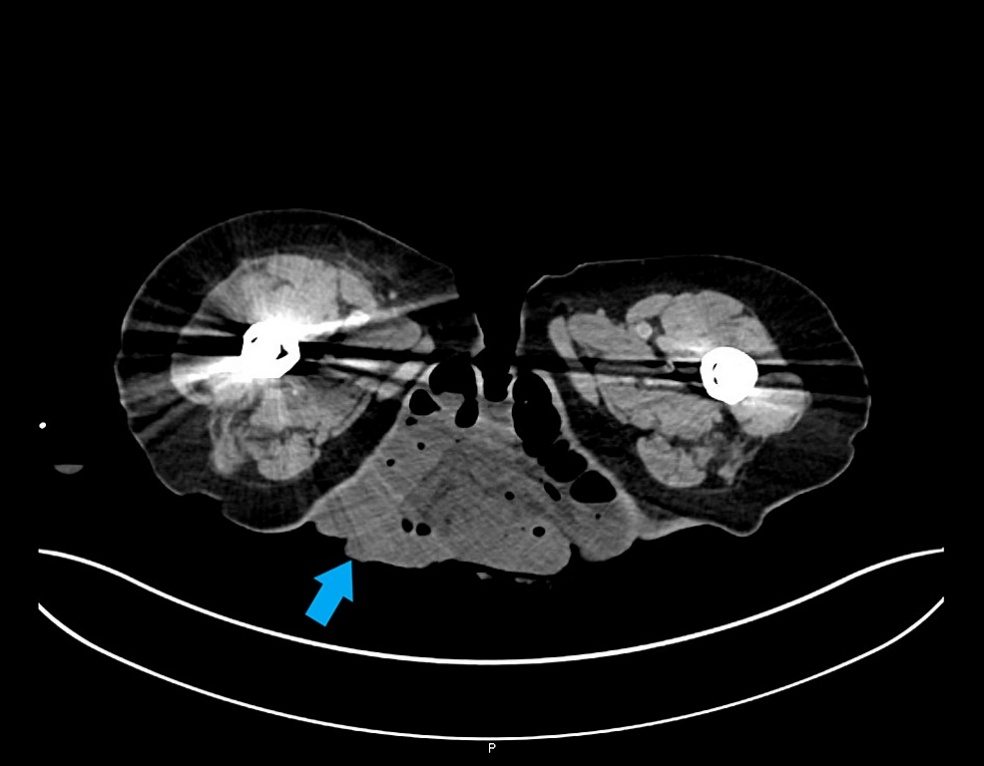
Axial CT abdomen and pelvis scan, showing the external herniated loops of small bowel.

Blood results on admission showed lactate of 1.8 mmol/L, C-reactive protein (CRP) 27 mg/L, haemoglobin (Hb) 117 g/L, and white cell count (WCC) 17.1 x 10^9^/L. The patient’s background of chronic kidney disease was considered when assessing her renal function, which demonstrated urea 12.7 mmol/L, creatinine 141 μmol/L, and estimated glomerular filtration rate (eGFR) 27 mL/min/1.73m^2^. Vital observations remained stable. A CT scan of the abdomen and pelvis (Figure 2) showed that the herniated small bowel was mildly dilated compared to the remaining intra-abdominal loops.

The patient was commenced on intravenous fluids and antibiotics, as per specialist microbiology advice. Urgent intervention was performed in the emergency department: the patient was placed in Trendelenburg position and safely sedated by the anaesthetist for manual reduction of the eviscerated small bowel into the abdominal cavity through a 4 cm to 5 cm defect in the posterior fornix.

As the patient had presented to the hospital in the late evening and on a weekend, a multi-disciplinary discussion between the colorectal, gynaecology and anaesthetic teams came to an agreement on the timing of surgery. After consideration of the limited theatre staffing and facilities at night, as well as the potential complexities of the case, it was deemed to be in the patient’s best interests of safety to be taken to theatre the following morning. The team was satisfied that the eviscerated loops of the bowel had no sign of ischaemia or necrosis. The reduced small bowel was kept in place overnight by using a firm aqueous Betadine-soaked vaginal gauze pack, and the patient remained in Trendelenburg position.

The following day, a lower midline exploratory laparotomy was performed. Adhesions of the small bowel were divided, and inspection of the small bowel demonstrated some inflammatory reaction with fibrin deposits. The bowel was not compromised, and as such, resection was not necessary. There was a 4 cm gap at the vaginal vault, but no abnormality of an infective or malignant nature was found. The vaginal vault was therefore repaired trans-abdominally with 2-0 polydioxanone (PDS) in two layers.

The patient was transferred to the surgical high dependency unit for post-operative observation. She received a blood transfusion and management of bradycardia and oliguria with acute-on-chronic kidney injury. On post-operative day 2, she was moved to the general surgical ward for step down of care and placed on a soft oral diet. The patient was passing flatus and documented a large bowel movement on post-operative day 6 when she was reviewed as medically fit for discharge.

## Discussion

The incidence of transvaginal evisceration after hysterectomy has been reported as 0.28% but is likely to be higher [3]. Total laparoscopic hysterectomy may be associated with a higher incidence of vaginal cuff dehiscence than a total abdominal hysterectomy or vaginal hysterectomy [3]. The reported median times from hysterectomy to evisceration are three months following total laparoscopic, 6.5 months following abdominal, and 34 months following vaginal hysterectomy [4]. Patients may first present with pelvic pain or vaginal bleeding, or they may present with obvious protruding viscera from the vagina [2]. If there is a delay in recognition and management, there may be serious risks of morbidity or mortality for the patient.

The patient, in our case, was an elderly woman who had a history of total abdominal hysterectomy and previous repair of pelvic organ prolapse more than 15 years before this event. This is much later than the reported median time of 6.5 months from total abdominal hysterectomy to vaginal cuff dehiscence [4].

It is unknown when and how the vault defect occurred in our patient and if there was a specific trigger event leading to sudden bowel evisceration. The patient had longstanding problems with constipation and symptoms of acute abdominal pain before admission, and certainly, there would be increased intra-abdominal pressure with straining on defecation and vomiting. Overall, the patient was frail and elderly, with a history of recurrent falls, including a repeat fall on the morning of the presentation. It is unlikely that the falls have significance in this presentation unless penetrative trauma was sustained, and there is no evidence for this. On inspection during laparotomy, there were no identifiable causes for the 4 cm vault defect itself and no appearance of a malignant process.

We postulate that in this case, the small bowel eviscerated spontaneously from an increasingly weakened vaginal vault, with consideration of a weakened pelvic floor, previous vaginal surgery, and tissue atrophy in the post-menopausal state. This would have been compounded by secondary factors of acutely raised intra-abdominal pressures.

The decision for surgical management is taken by a multi-disciplinary team and is usually led by assessing bowel viability [5]. The exposed bowel and vaginal defect should be examined under anaesthesia and, where possible, reduced intraperitoneally through the vagina. If the attempt at a reduction is unsuccessful, or if there is any suspicion regarding the viability of the bowel, the patient should undergo emergency laparotomy for abdominal exploration, bowel reduction and primary repair of the vaginal defect [1]. Ischaemic, non-viable bowel would require resection and anastomosis. Patients should also receive prophylactic broad-spectrum antibiotics to prevent infection from exposure of intra-abdominal contents to vaginal pathogens [5].

As an alternative to the abdominal approach, transvaginal evisceration can be managed by a vaginal and/or laparoscopic approach [4]. The vaginal approach may be appropriate if there is no sign of peritonitis, ischaemic injury, or strangulation, and the bowel can be replaced fully into the abdominal cavity through the vagina; this, however, limits thorough inspection of the bowel length. A combined laparoscopic and vaginal approach, on the other hand, enables appropriate inspection of the abdomino-pelvic viscera before repair of the vault defect (laparoscopically or vaginally) while also reducing surgical morbidity associated with laparotomy [5].

## Conclusions

Transvaginal evisceration of the small bowel should be recognised as a surgical emergency and managed by a multi-disciplinary team to prevent serious consequences, such as bowel ischaemia leading to resection, bowel necrosis, sepsis, and death. An immediate and thorough assessment of the eviscerated bowel should be undertaken to decide the appropriate route of surgical repair. If there is no doubt about the viability of the bowel, then it is feasible to undertake reduction and complete the repair vaginally. A combination of abdominal (open or laparoscopic) and vaginal surgical approaches allows for a thorough inspection of the length of the bowel and the nature of the vault defect before repair.
